# Cell-Based Therapies Formulations: Unintended components

**DOI:** 10.1208/s12248-016-9935-9

**Published:** 2016-05-27

**Authors:** Fouad Atouf

**Affiliations:** United States Pharmacopeial Convention, Rockville, Maryland USA

**Keywords:** ancillary material, cell-based therapy, excipient, formulation

## Abstract

Cell-based therapy is the fastest growing segment of regenerative medicine, a field that promises to cure diseases not treated by other small molecules or biological drugs. The use of living cells as the active medicinal ingredient present great opportunities to deliver treatment that can trigger the body’s own capacity to regenerate damaged or diseased tissue. Some of the challenges in controlling the quality of the finished cell-therapy product relate to the use of a variety of raw materials including excipients, process aids, and growth promotion factors. The quality of these materials is critical for ensuring the safety and quality of the finished therapeutic products. This review will discuss some of the challenges and opportunities associated with the qualification of excipients as well as that of the ancillary materials used in manufacturing.

## INTRODUCTION

Cell-therapy products offer new solutions to treat unmet medical needs by providing therapeutic products based on living cells that can promote the body’s own regenerative capacity. The cell-therapy market is one of the most diversified segments of the biotech industry; it includes human somatic and embryonic cells. A patient’s own (autologous) cells as well as donated (allogenic) cells or tissues can be used to manufacture cell-therapy products. In both cases, the development and manufacture of the finished product can require either minimal manipulation or extensive processing that can expose the cells to additional components. Major challenges in cell-therapy formulations are potential interactions between the components used in manufacturing and formulation, and the living cells. As with other biologics, cell-based therapy formulations include a variety of excipients (buffers, salts, polymers, proteins, and preservatives) that are added to stabilize the cells or to provide physiological osmolality. The manufacture of cell-based therapies often requires multiple steps where the cells are exposed to a variety of cell culture supplements not intended for inclusion in the final product. These cell culture supplements are referred to as process aids, ancillary reagents, or ancillary materials (1); the final harvest of cells prior to formulation and patient use may carry residual amounts of these materials. In this review, the term “excipients” will refer to components used in the formulation and to ancillary materials that may remain in the final product. The goal of this review is to provide an overview of the types of excipients used in cell-therapy formulations, as well as discuss risk assessment strategies to ensure their quality, and how to mitigate their impact on the critical quality attributes of finished cell-therapy products.

### Unintended Components

In the USA, the U.S. Food and Drug Administration (FDA) Guidance on Content and Review of Chemistry, Manufacturing, and Control (CMC) Information for Human Somatic Cell Therapy Investigational New Drug Applications (INDs) (2) states that “an excipient is any component that is intended to be part of the final product, such as human serum albumin or Dimethyl Sulfoxide (DMSO).” The US code of federal regulations require that all excipients used during the manufacture of products that are intended to be present in the final product must be listed in the Investigational New Drug (IND) Application (21 CFR 312.23(a) (7) (iv) (*b*)). The qualification of intended excipients present in the finished therapeutic product is required per 21 CFR 211.84(a). The United States Pharmacopeia’s book of public standards (referred to as a “compendia”), *U.S. Pharmacopeia–National Formulary* (*USP-NF*), defines excipients under General Chapter <1078> as follows: “Any substances, other than the active drug or product, that have been appropriately evaluated for safety and are included in a drug delivery system to either aid the processing of the drug delivery system during its manufacture, protect, support or enhance stability, bioavailability, or patient acceptability, assist in product identification, or enhance any other attribute of the overall safety and effectiveness of the drug delivery system during storage or use.” While excipients that are intended to be used in cell-therapy formulations that meet the definition will be qualified and tested against compendial monographs when they exist, some of the materials used in manufacturing other than the excipients may end up in the finished products. These ancillary materials are used as process aids for the manufacture of the cell-based therapies and for which removal from finished products may not be consistently effective. Sakamoto *et al*. (3) reported antibody responses in patients treated with cell-based therapies cultured in the presence of fetal calf serum and that bovine apolipoprotein B-100 is the predominant target of the antibody response. This observation suggests that the washing steps leading to the final harvest of a cell culture-based product may not be effective in clearing the cell suspension from proteins or other components in the media, which can be considered “unintended” components. Ancillary materials should go through the same risk assessment for inclusion as the excipients used in the product. Risk based approaches should take into account supplier qualifications, control of supply chain and proper documentation, and testing strategies. Such approaches will allow the identification of critical ancillary materials so that they can be qualified and sourced as for the excipients. Ancillary materials used in large quantities, materials used downstream of the process that may come in contact with the cells prior to final harvest, and complex mixtures such as animal-derived materials are examples of high-risk materials that are more critical.

### Quality of Components and Requirements for Novel Formulations

The excipients and other materials intended for use in cell-based formulations may have been qualified for use in other drug and biologic formulations. Table I shows examples of US approved cell-therapy products with a list of reported inactive ingredients (excipients) in addition to other ingredients used as ancillary materials with the potential to be present in the final formulation at residual levels. The reported inactive ingredients are known excipients with established safety profiles. However, applying formulations and excipients shown to be successful for existing dosage forms to new finished cell-therapy products requires a scientific justification for the use of each excipient in addition to toxicity studies to demonstrate that the excipient does not have deleterious effects on living cells. The use of established excipients in cell-therapy formulations will require evaluating the potential toxicity of the excipient on the cells, if the excipient is used a higher amount or in a new route of administration and will also require bridging safety and toxicological studies. Additionally, the potential impact of the cells and cell culture byproducts on the functionality of the excipient needs to be addressed. A scientific rationale needs to be provided for the introduction of novel excipients, demonstrating how the novel excipient improves the stability, safety, or efficacy of the finished product. The FDA Guidance for Industry on Nonclinical Studies for the Safety Evaluation of Pharmaceutical Excipients (4) provides guidelines on the toxicity information that must be submitted to the FDA in support of the use of a new excipient. Additional information on regulatory requirements can be found in the International Conference on Harmonisation of Technical Requirements for Registration of Pharmaceuticals for Human Use (ICH) Q8 Pharmaceutical Development (R2) (5) where it states that “the ability of excipients to provide their intended functionality, and to perform throughout the intended drug product shelf life, should also be demonstrated.” An example of repurposing an established excipient for cell therapies is the use of poloxamer based hydrogels as cell carriers, which has gained attraction with some of the cell-based regenerative medicine products under development (e.g., therapies to treat bone defects). Poloxamers have been established as pharmaceutical excipients in a variety of topical dosage formations including dental and burn dressing applications (6) Some poloxamers are water-soluble and will form a gel at higher temperatures (e.g., body temperature) and can be used to deliver cells into the human body as an alternative to solid scaffolds. During development of such products, the safety profile of and experience with these types of gels may be used and extrapolated to cell- and tissue-based therapies; however, the impact of these materials on living cells (and *vice versa*) needs to be evaluated.

**Table I:** Examples of Excipients and Formulations in the US Licensed Cell Therapy Products

Product name	Manufacturer	Active ingredient and reported inactive ingredients^a^	Description cell type and formulation^a^	Additional product description and potential ingredients that may end up in the product^a^
Provenge ® (autologous)	Dendreon	*Sipuleucel-T* *Inactive Ingredients:* Calcium chloride Potassium chloride Sodium chloride Sodium lactate Water	CD54+ cells activated with prostatic acid phosphatase (PAP) linked to granulocyte-macrophage colony-stimulating factor (PAP-GM-CSF) in Lactated Ringer’s Injection, USP	Peripheral blood mononuclear cells, including antigen presenting cells (APCs), activated with a recombinant human protein, PAP-GM-CSF,
Laviv ™ (autologous)	Fibrocell Technologies, Inc.	*Azficel-t*	Cultured dermal fibroblasts suspended in Dulbecco’s Modified Eagle Medium without Phenol Red.	Cells are expanded and then cryopreserved in a protein-free solution containing DMSO. Cells are thawed, washed, and shipped to the clinic.
Carticel® (autologous)	Genzyme Biosurgery	*Autologous cultured chondrocytes*	Cultured chondrocytes cells aseptically processed and suspended in 0.4 mL of sterile, buffered Dulbecco’s Modified Eagles Medium (DMEM).	Cells expanded in media containing 50 μg/mL gentamicin. Residual quantities of gentamicin up to 5 μg/mL may be present in the Carticel product
Gintuit (allogeneic)	Organogenesis	*Foreskin fibroblast,* *bovine type I collagen, and* *foreskin keratinocyte (neonatal)*	Cultured keratinocytes and fibroblasts in bovine collagen for topical application in the oral cavity.	Cells shipped in an agarose gel medium to maintain product potency and therefore may contain low amounts of inactive components present from the media. These include agarose type IV HI EEO, l-glutamine, hydrocortisone, full-chain human recombinant insulin, ethanolamine, O-phosphorylethanolamine, adenine, selenious acid, Dulbecco’s Modified Eagle Medium (DMEM) nutrients, Ham’s F-12 nutrients, sodium bicarbonate, calcium chloride, and water for injection.
ALLOCORD (allogeneic)	SSM Cardinal Glennon Children’s Medical Center St. Louis Cord Blood Bank	*Human cord blood Hematopoietic progenitor cell* *Inactive ingredients:* *Dimethyl sulfoxide (DMSO)* *Dextran 40*	The active ingredient is hematopoietic progenitor cells which express the cell surface marker CD34. The cellular composition depends on the composition of cells in the blood recovered from the umbilical cord and placenta of the donor.	When prepared for infusion according to instructions, the infusate contains the following inactive ingredients: PrepaCyte-CB separation solution, citrate-phosphate-dextrose, Dextran 40, human serum albumin, and residual DMSO.

^*a*^Source of information: www.dailymed.nlm.nih.gov

### Risk Assessment Strategies and Qualification of Components

The use of established excipients is the preferred formulation approach. However, this is not always an option and developers of cell-therapy products will often use non-pharmaceutical grade materials. In these situations, strategies need to be in place to control the safety and quality of all raw materials used in manufacturing. Risk assessment must address the complexity and multicomponent nature of the raw materials. Animal-derived materials are considered to be a higher risk because of the potential of contamination with adventitious agents, and justification is needed for the use of such materials, as well as testing to confirm absence of adventitious agents. Additionally, information on the country of origin is required per Good Manufacturing Practices (GMPs) regulations when using materials that carry the risk of transmissible spongiform encephalopathy. The use of human-derived materials in cell-based therapies carries the risk of transmission of communicable diseases. In line with the quality risk management described in ICH Q9, a risk mitigation strategy should include having controls in place to assess the impact of using animal- or human-derived excipients. While pharmaceutical-grade excipients are considered low risk because of their established safety profile, it is important to note that suitability of the excipient and its impact on the therapeutic effect of the cells need to be established. A strong qualification program is required to ensure the quality of excipients and other components used in the manufacture of cell-therapy formulations. Proper specifications are required to determine the suitability of these components during manufacture and in the final formulation. The manufacturing history of these components as well as the vendor (preferably the manufacturer) needs to be documented. The relationship between the cell density and the final concentration of the excipient needs to support the intended use of the excipient. The stability of the excipients should be assessed in the presence and absence of the actual cell-therapy product given that the established stability profile of the excipient by itself may not be relevant once it is added to the formulation. It is also critical to determine if potential degradation of the excipient might have deleterious effects on the finished product. Overall, the stability and viability the cells and expression of therapeutic entity needs to be established. While the intent is to remove the non-cellular components from finished cell-therapy products, residual levels of ancillary materials may remain due to ineffective washing steps or because of uptake or binding to the cellular components. The development of robust assays to measure levels of ancillary materials in the final formulation is critical to show the effectiveness of their removal. If residual amounts of the ancillary materials are present in the finished product, they should be qualified including toxicity assessment in animal models or other systems.

### Cryopreservation and Formulations of Cell-Therapy Products

Cell-therapy products prepared for specific patients (e.g., autologous bone marrow derived cells) can be maintained in liquid suspensions or formulations until administration. For other cell-therapy applications, the timing between cell collection/processing and administration to the patient may require an additional hold time. In these situations, cryopreservation may be a suitable approach to maintain product stability during storage and shipping steps (7). Cryopreservation may also be applicable to preserve the starting cell-source material and intermediate products before further processing to manufacture the final product. In all applications, the goal is to ensure preservation of product characteristics and quality attributes. Additionally, for large-scale manufacturing of allogenic cell-therapy products, cryopreservation facilitates the establishment and implementation of quality systems and workflow optimization allowing timely lot-release testing, management of a product inventory, transport of the product to the clinical site, and coordination of product administration (8). While cryopreservation is critical for stabilizing cell-therapy products until administration to the patient, the components of a cryopreservation medium may impact the safety and efficacy of the finished products. Serum-free and protein-free cryopreservation media formulations are commonly used. While cell product washing methods prior to administration must be validated to ensure adequate recovery and cell functions and removal of cryopreservation components, it is important to note that for some of the applications, the product is delivered to the patient in the freeze media to minimize manipulation of the cryopreserved cells. Cell washing does not guarantee complete removal of cryopreservation components and residual levels may be detected in the finished product; their levels should be estimated or measured. In both scenarios, the cryopreservation media components may be accounted for as excipients and qualified as such. Dimethyl sulfoxide (DMSO) is commonly used in cryopreservation media for starting cellular materials such as hematopoietic stem cells derived from cord blood, which are used to manufacture autologous or allogenic cell products to treat some genetic disorders that affect the immune system. High levels of DMSO may be given to patients who receive multiple doses of or multiple cryopreserved cell-therapy products. Developers of these products will use compendial-grade DMSO as there are monographs for DMSO in both the US Pharmacopeia and European Pharmacopoeia. In addition to using DMSO manufactured under a Good Manufacturing Practice (GMP) environment and that meet compendial standards, cell-therapy manufacturers can reference a drug master file for DMSO filed with the US FDA. It is important to highlight that in addition to ensuring the quality of DMSO itself, special attention needs to be paid to the packaging material used for cryopreservation and formulation of the cell products. DMSO interacts with many polymeric materials, and only high-density polypropylene and polytetrafluoroethylene materials are suitable for packaging a product containing high levels of DMSO (9). Package testing and compatibility with DMSO should be built into the risk assessment strategy.

### Excipients and Quality of Finished Products

Among biologic drugs, cell and tissue therapies are unique in the sense that their “active ingredients” may be living cells capable of continuous growth and they can secrete metabolites, cytokines, and other growth factors in the media. This may increase the possibility for excipients to interact with media components, and cells and their functions may be impacted because of these interactions. It is therefore critical that a risk assessment strategy for the qualification of excipients used in cell-based therapies encompass elements beyond standard approaches for excipients used in drugs and other biologics. Cell-therapy manufacturers need to address the functionality and stability of the excipients used in these applications. The functionality of an excipient established within a formulation for another drug or biologic may or may not be extrapolated to a cell-therapy formulation and may require testing of multiple batches of the excipient in multiple batches of the cell-therapy product. Another challenge is multisource suppliers of the same grade of excipient which can lead to batch-to-batch or supplier-supplier variability and, potentially, inequivalent performance of the excipient. The successful manufacture of a robust product requires the use of well-defined excipients and processes that together yield a consistent product (USP <1078> (10), section of GMP principles). Among the factors that contribute to quality of finished products are the following: adherence to cGMPs; process control and validation; and appropriate testing and quality of components added to manufacturing. For cell-based therapies, the latter is even more critical as the components used in manufacturing come in intimate contact with the active ingredients—the cells—whether these are process materials or elements of final formulations. A robust qualification program for starting materials, ingredients, excipients, and other raw materials is important to ensure a successful manufacturing process and the safety and efficacy of finished products. Excipients and cell culture media components need to be carefully selected especially for complex and not well-defined substances (e.g., material of animal origin). Cell therapies are manufactured under aseptic conditions and generally are intended to be delivered through injection or infusion of cells. Like any other therapeutic produced by aseptic processing, cell-based therapies can be contaminated through components including microorganisms or endotoxins. The starting material, water for injection, excipients, and other raw materials are all potential sources of contamination. It is important to characterize the bioburden and measure the endotoxin levels of each component and establish appropriate acceptance limits. Sterility assurance and freedom from endotoxins are critical for cell- and tissue-based products more than other biologics because these products are not subject to treatments such as terminal sterilization and filtration.

## CONCLUSIONS

As cell-therapy applications are increasingly introduced to the market, qualification of ancillary materials used in manufacturing should be addressed as well as excipients because of the potential for residual ancillary material in the finished products. Regardless of whether the material is an excipient or an ancillary material, the functionality of the material needs to be addressed in the context of the formulation as these materials may interact with the living cells impacting their function (and *vice versa*) and that this impact may be further amplified by the batch-to-batch or supplier-supplier variability. The introduction of novel excipients or other elements in the formulation of cell-therapy products must take into account and address the possible impact on the quality of the finished products. Cryopreservation media components must be evaluated in order to maximize the therapeutic efficacy of the cells. Components of formulations used in cell therapies must be of the highest quality to ensure consistency in manufacturing and quality and safety of finished medicinal products. Quality of these components may be achieved by testing against established public standards such as USP standards for excipients and ancillary materials.
