# Correction to: Cell-Based Therapies Formulations: Unintended Components

**DOI:** 10.1208/s12248-022-00747-5

**Published:** 2022-08-30

**Authors:** Fouad Atouf

**Affiliations:** United States Pharmacopeial Convention, Rockville, Maryland USA

**Correction to: The AAPS Journal, Vol. 18, No. 4, July 2016 (# 2016)**

**https://doi.org/10.1208/s12248-016-9935-9**

The article Cell-Based Therapies Formulations: Unintended components written by Fouad Atouf, was originally published Online First without Open Access. After publication in volume 18, issue 4, page 844-848 the author decided to opt for Open Choice and to make the article an Open Access publication. Therefore, the copyright of the article has been changed to © The Author(s) 2016 and the article is forthwith distributed under the terms of the Creative Commons Attribution Creative Commons Attribution 4.0 International License, which permits use, sharing, adaptation, distribution and reproduction in any medium or format, as long as you give appropriate credit to the original author(s) and the source, provide a link to the Creative Commons licence, and indicate if changes were made. The images or other third party material in this article are included in the article’s Creative Commons licence, unless indicated otherwise in a credit line to the material. If material is not included in the article’s Creative Commons licence and your intended use is not permitted by statutory regulation or exceeds the permitted use, you will need to obtain permission directly from the copyright holder. To view a copy of this licence, visit http://creativecommons.org/licenses/by/4.0/.

